# DNA damage response (DDR) and senescence: shuttled inflamma-miRNAs on the stage of inflamm-aging

**DOI:** 10.18632/oncotarget.5899

**Published:** 2015-09-29

**Authors:** Fabiola Olivieri, Maria Cristina Albertini, Monia Orciani, Artan Ceka, Monica Cricca, Antonio Domenico Procopio, Massimiliano Bonafè

**Affiliations:** ^1^ Department of Clinical and Molecular Sciences (DISCLIMO), Università Politecnica delle Marche, Ancona, Italy; ^2^ Center of Clinical Pathology and Innovative Therapy, Italian National Research Center on Aging, INRCA-IRCCS, Ancona, Italy; ^3^ Department of Biomolecular Sciences, Biochemistry and Molecular Biology, Università degli Studi di Urbino “Carlo Bo”, Urbino, Italy; ^4^ Department of Experimental, Diagnostic and Specialty Medicine, DIMES, University of Bologna, Bologna, Italy

**Keywords:** microRNA, senescence-associated secretory phenotype, senescence, inflamm-aging, Gerotarget

## Abstract

A major issue in aging research is how cellular phenomena affect aging at the systemic level. Emerging evidence suggests that DNA damage response (DDR) signaling is a key mechanism linking DNA damage accumulation, cell senescence, and organism aging. DDR activation in senescent cells promotes acquisition of a proinflammatory secretory phenotype (SASP), which in turn elicits DDR and SASP activation in neighboring cells, thereby creating a proinflammatory environment extending at the local and eventually the systemic level. DDR activation is triggered by genomic lesions as well as emerging bacterial and viral metagenomes. Therefore, the buildup of cells with an activated DDR probably fuels inflamm-aging and predisposes to the development of the major age-related diseases (ARDs). Micro (mi)-RNAs - non-coding RNAs involved in gene expression modulation - are released locally and systemically by a variety of shuttles (exosomes, lipoproteins, proteins) that likely affect the efficiency of their biological effects. Here we suggest that some miRNAs, previously found to be associated with inflammation and senescence - miR-146, miR-155, and miR-21 - play a central role in the interplay among DDR, cell senescence and inflamm-aging. The identification of the functions of shuttled senescence-associated miRNAs is expected to shed light on the aging process and on how to delay ARD development.

## INTRODUCTION

The DNA damage response (DDR) is an evolutionarily conserved signaling cascade, activated by DNA damage, which directs cell fate toward DNA repair, senescence, or apoptosis [1]. In higher organisms the DDR is thought to prevent neoplastic transformation in a cell-autonomous manner, by ensuring removal of severely damaged cells [2]. However, emerging data suggest that DDR signaling can also work through a paracrine/systemic mechanism, shaping the systemic environment through regulation of tissue repair and immune responses. Persistent DNA damage signaling (*i.e.* telomere attrition) may result in the DDR sending “early” and “late” extracellular signals, and in the induction of a senescence-associated secretory phenotype (SASP) [1, 2, 3]. DDR/SASP signaling involves a variety of biologically active proinflammatory mediators, including interleukins, chemokines, growth factors, matrix-degrading enzymes, and reactive oxygen species (ROS) [4]. Its role in the inflammatory response to tissue damage is epitomized by the observation that the major factors involved the setting up of the secretome are the proinflammatory transcription nuclear factor (NF)-kappaB (NF-kB) and the inflammasome [5, 6, 7]. NF-kB transcriptionally induces a variety of inflammatory SASP components (*e.g*. interleukin [IL]-6, IL-1 and tumor necrosis factor [TNF-α]), which are essential cell-autonomous regulators of senescence [3, 8, 9, 10, 11]. The SASP components, under the control of the inflammasome, are also able to propagate paracrine senescence to neighboring cells, which become capable of acquiring the SASP phenotype [7]. Senescence can thus spread “senescent cell” by “senescent cell”, at the tissue and the systemic level [12]. The identification and characterization of all DDR/SASP secretome components is thus expected to provide valuable information on the aging process and on how to delay the development of age-related diseases (ARDs). It is reasonable to hypothesize that the age-related increase in the burden of cells with DDR/SASP activation links cell senescence and inflamm-aging, and that non-coding RNA, mainly microRNAs (miRNAs), play a key role in the diffusion of DDR/SASP signaling to surrounding non-damaged cells during human aging, suggesting that the identification of new DDR/SASP signaling components may lead to develop novel therapeutic interventions against ARDs.

### Systemic spread of genomic damage: inflamm-aging

Senescence is a distinctive phenotype of eukaryotic cells involving the loss of replication ability and the acquisition of characteristic features - such as flattening and increased β-galactosidase (SA-β-gal) and p16INK expression - in response to a variety of stimuli that induce DNA damage, including extensive *in vitro* replication [13]. Senescence *per se* has long been known to be a mechanism halting the replication of cells that have acquired potentially hazardous genetic mutations [2, 14]. The finding that late-life clearance of senescent cells in a progeroid mouse model attenuates the progression of already established ARDs lends support to the notion that cell senescence is crucially involved in aging [15]. Notably, the same result has been achieved using a combination of molecules (*e.g.* quercetin and tyrosine kinase inhibitors), confirming the feasibility of selective senescent cell ablation and the effectiveness of senolytic drugs in alleviating symptoms of frailty and in extending health-span [16]. Even though the buildup in normal aged tissues of overtly senescent cells has proved difficult to demonstrate, it appears to have recently been documented in animal models and human tissues. Indeed, an accumulation of SA-β-gal/p16INK-positive cells has been described in atherosclerotic plaques, peritumor stroma, endothelia exposed to shear stress, in wounds in non-physiological and pathological conditions [17], in astrocytes of patients with Alzheimer's disease [18], and in kidney [19], and skin of old individuals [20]. Notably, the recent, seminal demonstration that DNA damage alone can induce distinct aging phenotypes in mouse liver has provided new insights into the causative role of DDR as a driver of aging [21].

The finding that the DDR is associated with SASP acquisition has further documented the complex relationship among DDR, cellular senescence, aging and ARD development [22, 23]. Even though “atypical” senescent states may arise independent of DDR activation [24], a wealth of evidence demonstrates that SASP is under the control of the DDR machinery [13, 25]. Conceivably, the physiological role of SASP is to act as an alarm system triggering the recruitment of immune cells (*i.e.* NK cells), to clear senescent/damaged cells from tissues [26]. Indeed, the SASP is viewed as an evolutionarily conserved, molecular tissue homeostasis program [27] that exerts beneficial early in life [28]. In adulthood it is held to modulate the remodeling and repair of damaged tissues and to promote the clearance of damaged/senescent cells through activation of innate immune cells [29] Notably, the spread of senescence among ”bystander cells” requires DDR activation [30], suggesting that the DDR and the ensuing inflammatory response are crucially involved in the propagation of aging phenotypes at the tissue and systemic levels. The notion is reminiscent of the so called “radiation-induced” bystander effect, where soluble factors from cells exposed to ionizing radiation (IR) or radioactive particles have been seen to activate the DDR machinery in non-exposed cells [31, 32]. A variety of mediators, including inflammatory factors, and NF-kB activation have been implicated in the phenomenon [33, 34]. Recently, it has been suggested that the diffusion of the radiation-induced bystander effect mimics that of radiation-induced senescence [35]. Consequently, DDR activation in a small subset of cells, including stem cells, may be sufficient for local and systemic SASP propagation, fuelling of inflamm-aging, and facilitation of chronic ARD development [36].

### Metagenomic tailoring of inflamm-aging

DDR activation is critical for the replication of cytomegalovirus [37]. Herpes-viruses have long been implicated in a variety of ARDs and associated with mortality in elderly cohorts [38]. Indeed, a broad range of human DNA viruses, including papilloma-viruses, polyoma-viruses, and herpes-viruses, exploit DDR activation for their own replication [37, 39, 40]; given their high prevalence in adulthood, it is reasonable to argue that most aging individuals are exposed to these exogenous DDR inducers in the course of their life. Recent data obtained by high-throughput metagenomics indicate that hundreds of DNA viruses dwell in biological fluids from healthy individuals, suggesting that an extraordinary amount of potential DDR-inducing agents may accrue with aging [41]. Notably, bacteriophages hosted by the local bacterial flora and non human-tropic viruses take part to viral communities isolated from different tissues and body compartments [42-44]. Consequently, each individual's metagenomic fingerprint is likely determined by the surrounding environment and ecosystem [41]. This may also include the “atypical” large DNA viruses that infect unicellular eukaryotes (i.e. amebae) [45]. At least in principle, most components of this emerging human virome are not frankly pathogenic, except in extreme conditions, such as immunodepression [46-48]; indeed, some may have evolved to exert protective/symbiotic functions, acting as a sort of virobiota capable of shaping and stimulating the immune system [49, 50]. Interestingly, the immune response and its activation under impaired immune conditions have been proposed as immune senescence mechanisms [51]. The above considerations suggest that virome-driven DDR activation may provide a significant contribution to the buildup of senescent cells, shaping each individual's inflamm-aging trajectory.

### MiR-146, miR-21, and miR-155: three key players in the inflamm-aging scenario

A large number of miRNAs modulate DDR activation and can promote or inhibit senescence and SASP in physiological and pathological conditions [52]. However, evidence regarding miRNA release in connection with DDR activation and/or of SASP acquisition and, especially, information regarding specific SASP-related secreted miRNAs, is quite limited. It is nonetheless reasonable that DDR/SASP-related miRNAs would share some common features such as: i) differential expression in senescent and young cells, making them senescence-associated (SA)-miRNAs; ii) the ability to modulate inflammatory pathways, primarily the NF-kB pathway, making them inflamma-miRNAs; iii) differential expression in total plasma/serum or in microparticles/exosomes of patients with the major ARDs; and iv) significant modulation induced by bacterial and viral infections. In this regard, virus-synthesized miRNAs can themselves target NF-kB, suggesting that metagenome- and genome-driven mechanisms both converge on the host inflammatory response [53]. At least three miRNAs - miR-146a, miR-155 and miR-21 - are consistent with this scenario and may constitute an SA/inflamma-miRNA system that can affect the systemic proinflammatory status and exert adverse effects on the pathways and mechanisms involved in organismal homeostasis [54-57].

#### miR-146

MiRNAs of the 146 family, including miR-146a and miR-146b, share all the above features. MiR-146a is one of the major miRNAs involved in orchestrating immune and inflammatory signaling *via* modulation of NF-kB activation and targeting of IL-1 receptor associated kinase (IRAK1 and 2) and TNF receptor-associated factor 6 (TRAF6) [58]. Intriguingly, miR-146a is an NF-kB-responsive miRNA [59] modulated by NF-kB through binding to different domains in the gene promoter region [60]. Therefore, miR-146a is a well-established SASP-modulating miRNA [61]; it is also a key player in a negative feedback loop directed at restraining excessive synthesis and secretion of proinflammatory molecules, *i.e.* cytokines, chemokines, and other proinflammatory molecules, ensuring a balanced NF-kB expression in cells under different conditions [62]. Notably, its involvement in the control of macrophage activation and polarization, both in human and in animal models reinforces the notion that it is one of the master modulators of the systemic inflammatory status [63]. MiR-146a is an SA-miRNA: during cell senescence its intracellular expression significantly increases in endothelial cells [64], trabecular meshwork cells (HTM) [65], smooth muscle cells [66], and fibroblasts [67]. It is upregulated in macrophages from aged rats, resulting in an age-associated cell dysfunction [68], and has been shown to be significantly modulated in senescent human kidney epithelial cells as well as T cells [69].

It has been reported that cells undergoing senescence but not exhibiting a robust SASP did not show miR-146a/b upregulation, and that IL-1-α neutralizing antibodies abolished both miR-146a/b expression and IL-6 secretion [70]. It is not surprising that plasma miR-146a levels are modulated in patients with a number of ARDs, such as type 2 diabetes [71, 72], rheumatoid arthritis [73], some cancers [74], and Alzheimer's disease [75]. Despite these considerations, the association between miR-146a and human diseases is extremely complex. Since miR-146a participates in a negative feedback loop mainly aimed to curb inflammation, dynamic changes in its expression are expected in tissues with different degrees of inflammation. A strong confirmation that miR-146a is involved in the modulation of inflamm-aging and ARD development has come from animal models: knockout of the miR-146a gene in mice leads to malignancies [76], and miR-146a-deficient mice develop low-grade, chronic, systemic inflammation [77].

The role of miR-146a has also been investigated in relation to the modulation of the immune response to bacteria and viruses. MiR-146, as well as miR-155 and miR-21, are commonly affected during bacterial infection, like Mycobacterium tuberculosis infection, and contribute to the immune response [78, 79]. Notably, miR-146 and miR-155 are co-induced in many cell types in response to microbial lipopolysaccharides (LPSs) to feedback-repress LPS signaling through Toll-like receptor (TLR) 4 [80]. MiR-146a expression is also modulated during viral infections. Elevated miR-146a expression impairs the expression pattern of interferon (IFN)-β by targeting TRAF6 in human monocytes infected with Dengue virus [81]. Similarly, vesicular stomatitis virus (VSV) has been found to modulate miR-146a expression and to impair IFN production, inhibiting the innate antiviral immune response [82]. The exploitation of cellular miR-146a by Chikungunya virus (CHIKV) is involved in the modulation of the host antiviral immune response [83]. In addition, it has been reported that miR-146a upregulation by Japanese encephalitis virus (JEV) leads to suppression of NF-kB activity and disruption of antiviral Jak-STAT signaling, which helps the virus evade the cellular immune response [84]. Hepatitis B virus (HBV) promotes miR-146a expression through the NF-kB signaling pathway, and miR-146a upregulation reduces the expression of an important negative regulator of the complement alternative pathway, promoting liver inflammation [85].

All these data suggest that chronic miR-146a overexpression could curb inflammation in aseptic conditions, as in accumulation of senescent cells in tissues, whereas in presence of bacterial or viral infection its upregulation may favor pathogen survival, contributing to the immunodeficiency (immunosenescence) associated with aging.

#### miR-155

MiR-155 also shares the features of DDR/SASP-related miRNAs. Its expression in macrophages increases in response to LPS, TNF-α, and IFN-β [86]. Its serum levels have a diagnostic role in patients with a variety of carcinomas [87]. MiR-155 upregulation has been described in bone marrow-derived dendritic cells (BMDCs) under activating conditions [88]. Moreover, in human endothelial cells (HUVECs) it regulates the expression of several inflammatory molecules, attenuating the adhesion of Jurkat T cells to activated HUVECs and reducing HUVEC migration [89]. In addition, miR-155 can inhibit IR-induced senescence both by acting downstream of the p53 and p38 mitogen-activated protein kinase (MAPK) pathways and by regulating tumor protein 53-induced nuclear protein 1 (TP53INP1) expression [90].

Notably, the recent report of a role for it as a key regulator of telomere stability has led to its inclusion among “telo-miRNAs” [91]. Surprisingly, miR-155 upregulation antagonizes telomere integrity and increases genomic instability. This finding suggests that miRNAs may have opposite effects on senescence, promoting or protecting from cell senescence based on the differential expression of their target in receiving cells: in cells involved in the inflammatory response miR-155 seems to have beneficial effects by targeting pathways that participate in the modulation of inflammation, whereas in those not directly involved in inflammation it may promote genomic instability and cell senescence, thus contributing to accelerating aging and ARD development.

Furthermore, miR-155 modulates the response to a number of bacterial infections. Following mycobacterium tuberculosis infection, miR-155-deficient mice died significantly earlier than wild-type mice [92]. MiR-155, miR-146b, and their predicted target gene IL-6, are upregulated in *Helicobacter pylori*-positive gastroduodenal ulcer [93]. MiR-155 also modulates viral infections, such as HBV replication [94]. Notably, some viral miRNAs share seed sequence homology with human miR-155 [95, 96]. Both miR-155 and virus-encoded miR-155 orthologs regulate the expression of TLR3, a TLR family member that recognizes double-stranded RNA carried by some viruses such as retroviruses. Upon recognition, TLR3 induces INF production, which signals other cells to increase their antiviral defenses [97].

Unexpectedly, miR-155-deficient mice are resistant to autoimmune diseases [98, 99]. Upon stimulation with ATP, miR-155^−/−^ dendritic cells showed limited Th2 priming capacity and reduced chemotaxis and IL-1 β secretion [100].

Notably, increased miR-155 expression levels have recently been detected in human adipocytes and macrophages and in their supernatants under LPS stimulation, providing one of the first demonstrations that miR-155 is a component of the secretome involved in the modulation of inflammation [101].

On the whole, these data show a complex interplay between miR-155 and immunity, suggesting that this miRNA may have different functions in innate and adaptive immune responses and that the systemic diffusion of this DDR/SASP-related miRNA may have both adverse and beneficial effects, depending on overall senescence/immunological host condition.

#### MiR-21

MiR-21 is another DDR/SASP-related miRNA candidate. Its function is especially complex, since demonstration of its aberrant expression in numerous cancers has led to its designation as an ‘onco-miR’; nonetheless it has also been shown as a key modulator in many inflammatory pathways [102, 103]. MiR-21 targets two important factors in the TLR signaling pathway, myeloid differentiation factor 88 (MyD88) and interleukin-1 receptor-associated kinase 1 (IRAK1). Its upregulation reduces replicative lifespan, while stable knock-down extends the replicative life span of normal endothelial cells [104]. A mathematical model integrating miR-21 and miR-146 expression into a signaling pathway in an *in silico* inflammation model has shown that the negative feedback provided by miR-21 stimulates the propensity of oscillations in NF-κB and IL-6 activity, whereas the negative feedback provided by miR-146a dampens them [105]. This process is fairly sensitive to the inputs of miR-21 and miR-146, suggesting that variations in the relative strength of the two feedbacks may provide for altered response dynamics to the same stimulus. The model may be applied to other inflamma-miR combinations. As expected for a putative DDR/SASP-related miRNA, miR-21 is upregulated during hepatitis C virus infection and negatively regulates IFN-α signaling through MyD88 and IRAK1; it may thus be a potential therapeutic target for antiviral intervention [106, 107].

#### Systemic miRNAs: a word about their shuttles

MiRNAs are released into the bloodstream inside vesicles, such as exosomes, microparticles and apoptotic bodies [108, 109], bound to HDL/LDL [110] or to RNA-binding proteins such as Argonaute 2 (Ago2) [111, 112].

Interestingly, views on the main miRNA shuttle strategies are discordant [113, 114]. Moreover, no data have been reported on the age-related prevalence of specific miRNAs and their shuttles. All miRNA transport strategies described to date allow communication between cells found in different organs. Exosomes may be the simplest and most robust way to realize a systemic miRNA-based signal network [115]. However, little is known of how miRNA species are sorted into exosomes and what miRNA binding proteins are involved.

Exosomes and microparticles released by irradiated cells have been demonstrated to reproduce the IR bystander effect [116], suggesting that DDR activation is capable of local and systemic signal diffusion and that miRNAs and their carriers may play a crucial role in it. An increased release of exosome-like microvesicles has been detected in normal human fibroblasts during replicative senescence or premature senescence [117]. Consistent with this finding, ceramide triggers exosome secretion, and endogenous ceramide levels increase with senescence onset [118, 119]. Moreover, treatment of young human endothelial cells with exogenous ceramide induces a senescent phenotype characterized by inhibition of cell proliferation and by a concomitant rise of SA-β-gal activity [120]. The increased ceramide biosynthesis seen during cellular senescence could thus contribute to increased exosome release demonstrated in senescent cells.

HDL is another well-established circulating miR carrier, shuttling the potent gene regulators to distant tissues. The report that miRNAs carried by HDL may be altered in disease states had further broadened our understanding of the complex effects that the lipoprotein can exert on target cells and tissues [121]. The delivery of lipid-associated miRNAs to recipient cells is achieved by various routes, including endocytotic uptake, membrane fusion, and scavenger receptors [122, 123].

**Figure 1:**
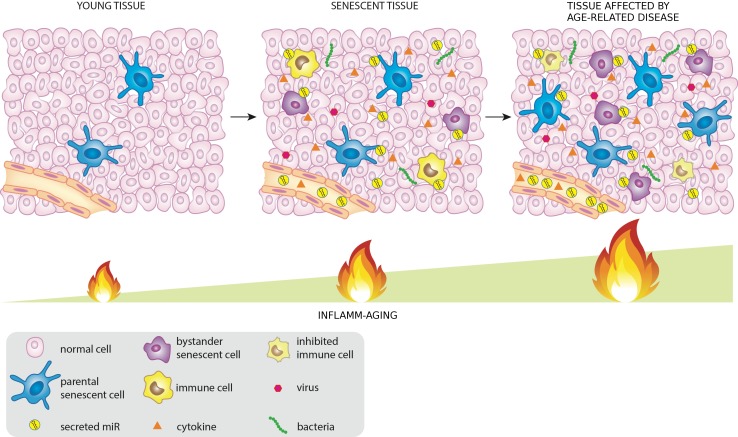
MiRNAs released by cells that activate the DDR/SASP may be involved in signaling to non-senescent cells, thus spreading inflamm-aging

It is reasonable to assume that a subset of miRNAs, released by senescent cells, infected cells or directly by pathogens (like viruses and bacteria) into the tissues and/or in the circulation inside exosomes or associated with HDL or binding proteins, may contribute to the systemic spread of DDR/SASP during aging modulating in turn both immunosenescence and inflamm-aging (Figure 1).

### miRNAs and their shuttles: servants of two masters

As noted above, bacterial and viral infections alter the endogenous miRNome and try to harness it to facilitate virus survival and replication in the host [124, 125]. A core temporal response to infection, shared across bacteria, has recently been described; it comprises a set of miRNAs, including miR-155, that may play an essential role in the regulation of basic cellular responses to stress [126]. Notably, DNA viruses encode viral miRNA, which can interfere with many cellular activities [127, 128, 129]. A relevant example is miR-155-like encoded by Kaposi sarcoma (KS) virus [95]. Moreover, viruses exploit exosomes to silence viral genes in latently infected cells, suggesting that the virus has evolved mechanisms that curtail rather than foster the spread of infection under certain conditions [128]. However, in some conditions exosomes released by infected cells may propagate the infection by evading the immune surveillance, primarily NK activation [127, 129]. Intriguingly, virus-promoted exosome biogenesis can also stimulate the immune system, eventually resulting in chronic stimulation promoting inflamm-aging- [130]. Notably, miR-21 and miR-146a seemed to be preferentially incorporated into exosomes and were virtually undetectable as free miRNAs in the supernatant of cells infected with Kaposi sarcoma-associated herpesvirus (KSHV) [131]. Transfer of the oncogenic exosomes to immortalized endothelial cells enhanced cell migration and IL-6 secretion, suggesting that KS-derived exosomes may be part of the paracrine signaling mechanism that mediates KSHV pathogenesis [131].

#### Emerging role of miRNAs and their shuttles in ARDs

**Figure 2:**
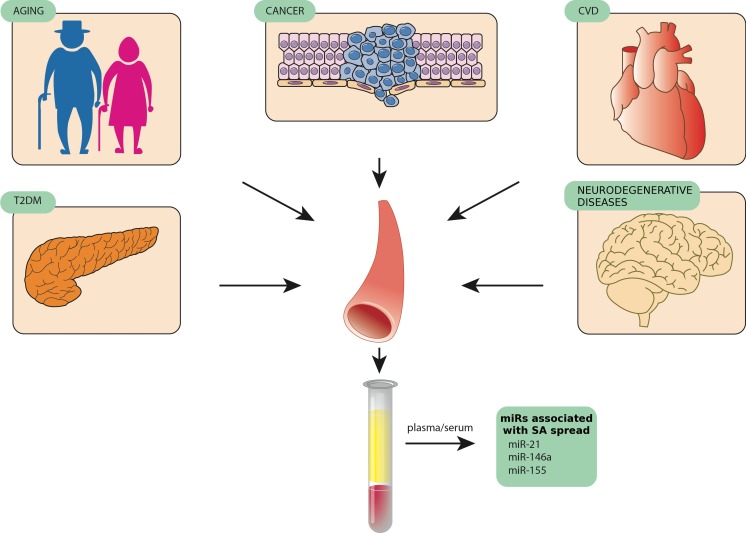
MiR-146, miR-155, and miR-21, three examples of circulating miRNAs released by different tissues in pathological conditions such as age-related diseases

MiR-21, miR-146a, and miR-155 are among the miRNAs being reported as biomarkers for a number of distinct human diseases, suggesting that they are involved in the modulation of non-specific pathogenic mechanisms [132]. The expression levels of these miRNAs are significantly modulated in senescent cells compared with younger ones as well as in plasma/serum of aged healthy subjects compared with patients with the most common ARDs [133-139]. SA-miRs and inflamma-miRs are modulated during normal aging, both in cells and biological fluids, and show differential expression in a number of ARDs, such as cardiovascular diseases, T2DM, autoimmune diseases, and cancers [140]. These findings support the emerging concept that senescent cells and their secretome have a broad biological significance in human physiological aging and in ARDs [141]. Indeed, specific targeting of senescent cells in different tissues has been demonstrated to delay ARD development, reducing chronic inflammation through SASP modulation and probably enhancing the appropriate immunomediated responses to pathogens [142]. Since an increasing number of studies have identified miRNAs associated with cellular aging and tissue degeneration [143-147], the miRNAs involved in inflammatory process and senescence control seem to be those best matching the “identikit” of DDR/SASP-related miRNAs that contribute to inflamm-aging (Figure 2).

## CONCLUSIONS

We have discussed the hypothesis that some miRNAs play a central role in DNA damage-related cell senescence and inflammaging. It is conceivable that miRNAs released by DDR/SASP-activating cells also signal to non-senescent cells, spreading and increasing inflamm-aging. A number of studies show that both senescent and infected cells can enact miRNA-based strategies to curb their own proinflammatory status. However, the contribution of senescent cells to release of the miRNAs involved in the modulation of systemic inflammation is not well explored; in particular, it is unclear whether changes in intracellular miRNA expression patterns during senescence are paralleled by changes in the patterns of released miRNAs. Nevertheless, it is conceivable that when senescent cells with an activated DDR/SASP exceed a given threshold, changes in the network of shuttle-associated miRNAs released in the bloodstream do ensue. Current evidence suggests that miRNAs may exert opposite effects, both promoting and protecting from cell senescence. The paradox may only be apparent, because miRNAs released by senescent cells may protect them from inflammation, but promote senescence in younger cells by targeting different mRNAs from those belonging to the inflammatory pathway.

Since miRNAs released in exosomes seem to be those with the greatest ability to interconnect cells in an endocrine manner, it can be hypothesized that miRNAs contained in exosomes are subject to senescence-associated modulation.

Identification of the profile of DDR/SASP-related miRNAs and their shuttles is expected to help clarify the intricate relationship between inflammation and immune surveillance in aging, and to lead to new therapeutic strategies that can reduce the risk of ARDs and delay their onset.
